# The frequency of HLA-DQ2/DQ8 haplotypes and celiac disease among the first-degree relatives of patients with celiac disease 

**Published:** 2021

**Authors:** Masoume Mansouri, Masoud Dadfar, Mohammad Rostami-Nejad, Golnaz Ekhlasi, Amirhossein Shahbazkhani, Bijan Shahbazkhani

**Affiliations:** 1 *Digestive Disease Research Center, Digestive Diseases Research Institute, Tehran University of Medical Sciences, Shariati Hospital, Tehran, Iran*; 2 *Health Center of Tarbiat Modares University, Tehran, Iran*; 3 *Gastroenterology and Liver Diseases Research Center, Research Institute for Gastroenterology and Liver Diseases, Shahid Beheshti University of Medical Sciences, Tehran, Iran*; 4 *Sina hospital, Tehran University of Medical Science, Tehran, Iran*

**Keywords:** Celiac disease, HLA-DQ2, HLA-DQ8, HLA typing, Iran

## Abstract

**Aim:**

We evaluated the frequency of human leukocyte antigen (HLA) DQ2/DQ8 haplotypes as well as celiac disease (CD) among the first-degree relatives (FDRs) of CD patients, compared with healthy controls, and compared the HLA typing with serologic tests in this population.

**Background:**

Until now, no study has examined the frequency of HLA-DQ2/DQ8 haplotypes among the FDRs of Iranian patients with CD.

**Methods:**

In the current case-control study, 100 FDRs of CD patients and 151 healthy controls were included. Demographic characteristics were assessed using a research-made questionnaire. A blood sample was collected from each participant for HLA-DQ typing and measuring serum levels of anti-gliadin and anti-transglutaminase (anti-tTG) antibodies.

**Results:**

The mean age of the FDRs of CD patients and controls was 30 and 35 years, respectively. Also, 51% (n=51) of the FDRs and 51.7% (n=78) of controls were female. CD was diagnosed among 3% (n=3) of the FDRs of CD patients. No significant difference was found in terms of the frequency of HLA-DQ alleles between the FDRs of CD patients and controls. Out of 100 FDRs of CD patients, 40% had HLA-DQ2 allele, 16% carried HLA-DQ8 allele, and 4% had both alleles. Surprisingly, the CD was diagnosed in three subjects among 60 FDRs of CD patients with HLA-DQ2 allele (3% of the whole population). This diagnosis was based on the results of serological tests as well as endoscopy and intestinal biopsy.

**Conclusion:**

CD was confirmed among 3% (n=3) of the FDRs of CD patients. We found that HLA typing is not effective in predicting CD among FDRs of CD patients. Other methods such as serological tests have a higher priority compared with HLA-DQ typing.

## Introduction

 Celiac disease (CD) is an autoimmune intestinal disorder that is triggered by the ingestion of gluten-containing foods including wheat, barley, and rye in genetically susceptible individuals (1,2). CD is multifactorial, but genetic factors have a major role in its etiology (3). Therefore, the family members of CD patients, especially first-degree relatives (FDRs), are at higher risk of developing CD than others (4). It is estimated that the frequency of CD among the FDRs of CD patients is from 5% to 38% (5,6). CD may be asymptomatic over many years and it may be diagnosed after 10 years since the first symptoms appear (7). Therefore, using precise and careful CD screening methods, particularly in high-risk asymptomatic individuals, is of great importance. 

The most important CD screening method is the anti-tissue-transglutaminase (anti-tTG) test (8). However, some studies have shown that human leukocyte antigen-DQ (HLA-DQ) typing can help predict CD in high-risk individuals such as FDRs of CD patients (9-11). Based on these studies, assessing HLA-DQ haplotypes including HLA-DQ2, HLA-DQ8, HLA-DQA1, and HLA-DQB1 alleles might be used for CD screening (9, 12, 13). The absence of HLA-DQ2 and HLA-DQ8 alleles can most likely rule out the presence of CD, while in the presence of these alleles, further screening methods such as CD-specific antibody testing are required for definite diagnosis of CD (9). 

Despite the frequent use of HLA typing for CD screening, its sensitivity and validity are questionable. Some researchers have claimed that HLA-DQ2/DQ8 typing can be used for the diagnosis of CD (7, 14), while others regard it as a good alternative for the detection of the subject’s genetic predisposition (12, 15). For instance, Kaukinen et al. reported that HLA-DQ2 and -DQ8 determination is useful in exclusion, probably lifelong, of CD in individuals with an equivocal small-bowel histological record (14). Studies that examined the frequency of HLA-DQ2/DQ8 haplotypes in high-risk individuals for CD are mainly from western nations and based on the literature search, with no study being conducted in Iran. In addition, there has been no research in this context to have examined the prevalence of CD in individuals with HLA-DQ2/DQ8 positive. Therefore, in this study, we aimed to evaluate the frequency of HLA-DQ2/DQ8 haplotypes as well as CD among the FDRs of CD patients, compared with healthy controls, and to compare the HLA typing with serologic testing in this population. 

## Methods

This case-control study was conducted in the Digestive Disease Research Center affiliated to the Tehran University of Medical Sciences, Tehran, Iran, from January 2014 to January 2015. Cases were FDRs of CD patients. In order to include cases, we selected some biopsy proven CD patients by reviewing medical records available in the Digestive Disease Research Center and then, contacted them to invite their FDRs (father, mother, siblings, and children) to the CD clinic at Shariati Hospital, Tehran, Iran. Exclusion criteria were the following: suffering from other gastrointestinal (GI) disorders, the presence of systemic disorders, and unwillingness to participate in the current examination. Overall, after considering inclusion and exclusion criteria, 100 FDRs of CD patients were included in the current study. In addition to cases, 151 healthy controls without any history of GI disorders were included in the current study. The research process and purposes were thoroughly explained to each participant and informed written consent was taken. Then, participants were asked to complete a research-made questionnaire on demographic characteristics including age, gender (male/female), relationship with the CD patient for cases (father/mother/ siblings/children), ethnicity (Fars/Tork/Lor/Baloch/Kord/Arab/Mazani/Afghani/etc), medical history, GI and non-GI symptoms and complaints (for FDRs). 

All participants signed an informed written consent before inclusion to clinical trial. All procedures performed in this study were in accordance with the ethical standards of the institutional and/or national research committee and with the 1964 Helsinki declaration and its later amendments or comparable ethical standards. The study was ethically approved by the ethics review board of the Digestive Disease Research Center, Digestive Disease Research Institute, Tehran University of Medical Sciences, Tehran, Iran, with the code IR.TUMS.DDRI. REC.1399.015. We caused no injury or damage to participants. The data collected from participants remained confidential. 

**Sample size calculation**

Sample size was calculated using Cochrane formula (16). By considering the type 1 error of 0.05 and the frequency of 74% for HLA-DQ2 allele among FDRs of CD patients, obtained from the study of Lopes et al. (17), the required cases (FDRs of CD patients) for this study were calculated 60 subjects. However, we enrolled 100 FDRs of CD patients in the present study. In addition, 151 healthy controls were included given the frequency of 42.6% for HLA-DQ2 allele among healthy individuals (18). 

**Sample collection and analysis**

A blood sample (10 cc) was taken from each participant and sent to Tehran Gholhak Laboratory for HLA typing. The blood sample was collected in ethylenediamino-tetra-acetic (EDTA) tubes and stored at -20 °C till further analysis. Genomic DNA was extracted from whole blood using the QIAamp DNA Blood Mini Kit (Qiagen, Hilden, Germany), according to the manufacturer’s instructions. Extracted DNA was stored at -20 °C until performing polymerase chain reaction (PCR). The genotyping to detect DQA1*05, DQB1*02, and DQB1*0302 alleles was performed based on SYBR Green PCR via the Olerup SSP™ PCR amplification kit (Genovision, Oslo). Then, the PCR products were detected by the electrophoresis, which were run on 2% agarose gel (Fluka Bio Chemica, Ronkonkoma, NY, USA) and finally viewed under the UV light (Bio-Rad). The frequency of HLA DQ2/DQ8 alleles was based on the frequency of the DQA1*05, DQB1*02, and DQB1*0302 alleles in genotyping. We considered DQ2-positive individuals as those who had the DQA1*05 and DQB1*02 alleles in genotyping and DQ8-positive subjects as those who had the DQB1*0302 allele.

To measure serum levels of anti-gliadin and anti-transglutaminase (anti-tTG) antibodies in those FDRs with HLA-DQ-positive, blood samples were centrifuged for 10 min at around 2500–3000. ELISA method with commercially available kits (Aesku. Diagnostics GmbH, Wendelsheim, Germany) was used to measure serum concentrations of anti-gliadin and anti-tTG antibodies. A normal range of tests was defined according to the laboratory standards and was finally reported as positive/negative. The Anti-gliadin levels of >10 RU/mL or anti-tTG levels of >20 RU/mL were considered as elevated levels or a positive result (19). After performing HLA typing and serological examinations, participants with positive results were referred to perform endoscopy and duodenal biopsy for a definite diagnosis of CD. 

**Statistical analysis**

Data analysis was performed using the Statistical Package for the Social Sciences (SPSS) version 19. Data were presented as number and percentage (%) for categorical variables and mean for continuous variables. To assess the distribution of individuals in terms of categorical variables between cases and controls and also across different types of HLA-DQ alleles, the Chi-square test was applied. The significance threshold was considered as P-value <0.05.

## Results

In the current study, 100 FDRs of 40 celiac patients and 151 healthy controls were included. Forty-two individuals were children or adolescences and others were adults. Of the 251 participants, 49% (n=122) were male and 51% (n=129) were female subjects. 

Differences in age, gender, and HLA-DQ alleles between FDRs of CD patients and controls are shown in Table 1. 

**Table 1:** Differences in age, gender, and HLA-DQ haplotypes between FDRs of CD patients and controls

		FDRs of CD patients (n=100)	Controls (n=151)	P-value
Age group (year)				0.01
	<22	23 (23.0)	19 (12.6)	
	22-44	61 (61.0)	88 (58.3)	
	>44	16 (16.0)	44 (29.1)	
Gender				0.91
	Male	49 (49.0)	73 (48.3)	
	Female	51 (51.0)	78 (51.7)	
HLA-DQ alleles				0.94
	HLA-DQ2	40 (40.0)	61 (40.4)	
	HLA-DQ8	16 (16.0)	21 (13.9)	
	HLA-DQ2/DQ8	4 (4.0)	8 (5.3)	
	Both negative	40 (40.0)	61 (40.4)	

Data are presented as number (percent). Abbreviations: HLA: human leukocyte antigen, FDRs: first-degree relatives, CD: celiac disease

**Table 2:** Demographic characteristics of the FDRs of CD patients across different types of HLA-DQ alleles

		HLA-DQ2 (n=40)	HLA-DQ8 (n=16)	HLA-DQ2/DQ8 (n=4)	Both negative (n=40)	P-value
Gender						0.27
	Female	18 (45.0)	6 (37.5)	2 (50.0)	25 (62.5)	
	Male	22 (55.0)	10 (62.5)	2 (50.0)	15 (37.5)	
Family relationship						0.70
	Father	11 (27.5)	5 (31.3)	1 (25.0)	6 (15.0)	
	Mother	10 (25.0)	3 (18.8)	1 (25.0)	13 (32.5)	
	Sister	3 (7.5)	4 (25.0)	1 (25.0)	11 (27.5)	
	Brother	8 (20.0)	2 (12.5)	1 (25.0)	7 (17.5)	
	Son	6 (15.0)	2 (12.5)	0	2 (5.0)	
	Daughter	2 (5.0)	0	0	1 (2.5)	
Ethnicity						0.40
	Fars	19 (47.5)	4 (25.0)	2 (50.0)	11 (27.5)	
	Kurd	2 (5.0)	0	0	2 (5.0)	
	Lur	2 (5.0)	0	0	4 (10.0)	
	Arab	0	0	0	1 (2.5)	
	Turk	9 (22.5)	9 (56.3)	1 (25.0)	12 (30.0)	
	Baluch	1 (2.5)	0	0	0	
	Gilakis	3 (7.5)	3 (18.8)	1 (25.0)	2 (5.0)	
	Mazandaranis	4 (10.0)	0	0	8 (20.0)	
Total		40 (100)	16 (100)	4 (100)	40 (100)	

Data are presented as number (percent). Abbreviations: HLA: human leukocyte antigen, FDRs: first-degree relatives

**Table 3:** Clinical symptoms of participants across different types of HLA-DQ alleles

Symptoms	HLA-DQ2	HLA-DQ8	HLA-DQ2/DQ8	Both negative
Abdominal pain	12 (30.0)	6 (40.0)	0	11 (27.5)
Diarrhea	4 (10.0)	2 (13.3)	0	1 (2.5)
Bloating	7 (17.5)	1 (6.7)	0	7 (17.5)
Aphtous	3 (7.5)	1 (6.3)	1 (25.0)	11 (27.5)
Constipation	9 (22.5)	1 (6.7)	3 (75.0)	6 (15.0)
Anorexia	4 (10.0)	3 (18.8)	1 (25.0)	3 (7.5)
Osteopenia	2 (5.0)	2 (12.5)	1 (25.0)	1 (2.5)
Anemia	19 (47.5)	6 (37.5)	0	19 (47.5)

Data are presented as number (percent). Abbreviations: HLA: human leukocyte antigen

**Table 4:** Information of three new cases of celiac disease among FDRs of previously known celiac patients

	Case 1	Case 2	Case 3
Family relationship	Mother of celiac patient	Mother of celiac patient	Mother of celiac patient
Age (mean)	53	32	44
Gender	Female	Female	Female
Ethnicity	Lur	Turk	Fars
HLA-DQ	DQ2 positive	DQ2 positive	DQ2 positive
Anti-tTG antibody (RU/mL)	210	210	205
Anti-gliadin antibody (RU/mL)	110	65	45
GI symptoms	Abdominal pain	Abdominal pain, bloating, constipation	No GI symptoms
Non-GI symptoms	Anemia	Anemia	Anemia, Aphtus ulcer

Abbreviation: HLA: human leukocyte antigen, GI: gastrointestinal, FDRs: first-degree relatives

No significant difference was found in terms of gender and HLA-DQ alleles between the FDRs and controls (P>0.90), but age was significantly different between the two groups (P=0.01). HLA typing showed that 60% (n=60) of the FDRs of CD patients carried HLA-DQ alleles, of whom 40% (n=40) carried HLA-DQ2 allele, 16% (n=16) had HLA-DQ8 allele, and 4% (n=4) carried both alleles. In controls, 59.6% (n=90) were HLA-DQ-positive, 40.4% (n=61) had HLA-DQ2 allele, 13.9% (n=21) had HLA-DQ8 allele, and 5.3% (n=8) had both alleles. 

The demographic characteristics of the FDRs of CD patients across different types of HLA-DQ alleles are shown in Table 2. Out of 40 FDRs with HLA-DQ2, 55% (n=22) were male and 45% (n=18) were female. In addition, 62.5% (n=10) and 37.5% (n=6) of the FDRs with HLA-DQ8 allele were male and female, respectively. Besides, 2 male and 2 female subjects had both alleles of HLA-DQ2 and HLA-DQ8. In terms of family relationships, HLA-DQ2 and HLA-DQ8 alleles were frequently detected among the fathers of CD patients such that 27.5% (n=11) of individuals with HLA-DQ2 and 31.3% (n=5) of those with HLA-DQ8 were the fathers of CD patients. Conversely, the distribution of HLA-DQ2 (n=2; 5%) and DQ8 (n=0) was at the lowest rate in the daughter of CD patients. With respect to ethnicity, most FDRs of CD patients with HLA-DQ2 allele were Fars (n=19; 47.5%) and then Turk (n=9; 22.5%), while most FDRs with HLA-DQ8 were Turk (n=9; 56.3%) and then Fars (n=4; 25.0%). FDRs with both alleles were mostly from Fars ethnicity (n=2; 50.0%). No significant difference was seen for the above-mentioned demographic variables across different types of HLA-DQ alleles (P>0.20). 

Clinical symptoms of FDRs of CD patients across different types of HLA-DQ alleles are presented in Table 3. The most prevalent symptoms among the FDRs with HLA-DQ2 or HLA-DQ8 allele were anemia (HLA-DQ2; n=19, 47.5%, HLA-DQ8; n=6, 37.5%) and abdominal pain (HLA-DQ2; n=12, 30%, HLA-DQ8; n=6, 40%), while those with both HLA-DQ2 and HLA-DQ8 mostly suffered from constipation (n=3, 75%). It should be kept in mind that the most common symptoms among FDRs of CD patients with HLA-DQ-negative were anemia (n=19, 47.5%) and abdominal pain (n=11, 27.5%).

Based on the results of serological tests, endoscopy and deodenal biopsy, the CD was diagnosed in three of 100 FDRs of the CD patients (3%). The information of these new cases of CD was illustrated in Table 4. All of these cases were female and were the mothers of CD patients. Furthermore, all had the HLA-DQ2 allele. The serum concentrations of anti-tTG antibody were positive (>20 RU/mL) in all new cases; however, only one patient was anti-gliadin-positive (>10 RU/mL). Of the mentioned three patients, all had anemia, two had abdominal pain, and one had no GI symptoms.

## Discussion

In the current study, no significant difference was found in terms of HLA-DQ alleles between the FDRs of CD patients and controls. We also found that 60% (n=60) of the FDRs of CD patients were HLA-DQ-positive, 40% (n=40) had HLA-DQ2 allele, 16% (n=16) carried HLA-DQ8 allele, and 4% (n=4) had both alleles. However, out of all FDRs of CD patients with HLA-DQ-positive, only 3 were diagnosed with CD (3% of the FDRs). To the best of our knowledge, the current study was the first to assess the frequency of HLA-DQ haplotypes among the FDRs of CD patients in Iran.

The prevalence of CD is increasing among adults (20). This is more important among FDRs of CD patients than the general population. In our study, CD was diagnosed in 3% of the FDRs of CD patients. Similar to our findings, Chomeili et al. showed that CD was diagnosed in 2 (6%) of 30 siblings of patients with confirmed CD (21). Genetic factors are involved in the etiology of CD and other GI disorders (22-25). HLA-DQ alleles have a high prevalence among CD patients (26). These alleles can determine the susceptibility of at-risk individuals such as the FDRs of CD patients (9). Previous studies revealed a high frequency of HLA-DQ2/DQ8 alleles among the FDRs of CD patients (27). However, to our knowledge, no study determined this frequency among Iranians. 

We found no significant difference between FDRs of CD patients and controls in terms of HLA-DQ alleles. Findings from the previous studies were not in line with our results. In a cross-sectional study in Brazil, HLA-DQ2/DQ8 was present in 98.4% of CD patients, 89.6% of the relatives of CD family, and in 55.4% of people from the general population without family celiac (28). Such a significant difference between HLA-DQ alleles between FDRs of CD patients and healthy individuals without family celiac was seen in the other two studies (11, 29). In a case-control study in Iran, Bahari et al. reported no significant difference between CD patients and their FDRs in terms of HLA alleles (30). The observed discrepancy might be due to the high prevalence of family marriage among Iranian population which can increase the rate of HLA-DQ haplotypes among general population. 

In the current study, 60% of the FDRs of CD patients were HLA-DQ positive, of whom40% had HLA-DQ2 allele, 16% carried HLA-DQ8 allele, and 4% had both alleles. In line with our study, a cross-sectional study on 89 FDRs of CD patients showed that 51.7% of FDRs were HLA-DQ2/DQ8 positive (11). In addition, researchers in that study concluded HLA-DQ2/DQ8 can be used to diagnose CD, particularly when the serological tests are useless to screen at-risk individuals. In a retrospective cohort study in the Netherlands, HLA-DQ2/DQ8 became positive among 87.5% of the FDRs of CD patients; among them, HLA-DQ2 homozygous sisters/daughters were at the highest risk of CD (31). The findings of that cohort indicated that one-time celiac-specific antibody testing alone could be sufficient to rule out the disease in at-risk individuals such as FDRs. In another study on 434 FDRs of CD patients, 87.4% had either HLA-DQ2 or -DQ8 alleles, while only 10.9% were diagnosed for CD (29). In a cross-sectional study in Spain, 78.4% of 139 FDRs of CD patients were HLA-DQ2/DQ8 positive (homozygous, 15.1%; heterozygous, 63.3%); however, GI symptoms were observed in 45.7% of the participants. In that study, despite negative serological markers, duodenal lymphocytosis and atrophy were frequently observed in the FDRs (32). 

Overall, it seems that HLA-DQ typing cannot be used for determining susceptible individuals to CD, neither can it be used for the diagnosis of CD. Of note, individuals with HLA-DQ-negative are less likely to have CD compared with those with HLA-DQ-positive. However, there is evidence indicating CD patients with HLA-DQ-negative. For instance, in the study of Rostami-Nejad et al., out of 51 CD patients, two were HLA-DQ-negative and others had different types of HLA-DQ alleles (33). In the current study, the CD was diagnosed in only three subjects among 60 individuals with HLA-DQ2/DQ8 positive. Altogether, due to the high cost of HLA-DQ typing and the low sensitivity of this method for the diagnosis of CD, it does not have sufficient priority compared with serological tests for screening CD in the Iranian population. 

In the current study, the role of different genetic backgrounds is well-reflected. We found that most subjects with HLA-DQ2 allele were Fars (47.5%) and then Turk (22.5%), whereas most individuals with HLA-DQ8 were Turk (56.3%) and then Fars (25.0%). Subjects with both alleles were mostly from Fars ethnicity (50.0%). The genetic implications of these differences must be evaluated and investigated extensively in future studies. The current investigation, despite its low sample size, was the first to determine the HLA-DQ alleles among the different ethnicities of the Iranian population. 

Generally, it is well-known that CD occurs in women more often than men with a ratio of around 2:1 (34, 35). As seen in the present study, three new cases of CD were female. However, in the analysis of HLA-DQ2 and HLA-DQ8 typing, the same pattern did not emerge. The frequency of HLA-DQ2/DQ8 alleles was higher in men than women. Despite the genetic predisposition in men, it seems that non-genetic factors such as diet contribute to the high prevalence of CD among women. Further investigations are needed to elucidate the cause of this gender difference in the frequency of CD and HLA-DQ haplotypes.

The current study was the first to examine the frequency of HLA-DQ haplotypes among the FDRs of CD patients in Iran. In addition, we assessed the frequency among men and women as well as across the different ethnicities of Iran. However, our study had some limitations. Due to limited financial resources, we could not perform serological tests for the participants with HLA-DQ-negative. In the current study, we could not assess other contributing factors to CD including diet and inflammatory biomarkers. In addition, the low sample size of our study and the lack of assessment of other CD-related genetic factors are other limitations of this study. Further studies by considering the limitations mentioned above are required in this regard. 

In conclusion, no significant difference was found in terms of HLA-DQ haplotypes between the FDRs of CD patients and controls. Out of 100 FDRs of CD patients, 40% had HLA-DQ2 allele, 16% carried HLA-DQ8 allele, and 4% had both alleles. Surprisingly, the CD was diagnosed in three subjects among 60 FDRs of CD patients with HLA-DQ2/DQ8. This diagnosis was based on the combined results of serological tests, endoscopy, and intestinal biopsy. Overall, given the high cost of HLA-DQ typing and its low sensitivity, we concluded that this method is not a good choice for the screening of CD among the Iranian population but serological tests have a higher priority compared with HLA-DQ typing. Nevertheless, further studies are needed to confirm our findings.

## Conflict of interests

The authors declare that they have no conflict of interest.
